# Reading Proficiency and Adaptability in Orthographic Processing: An Examination of the Effect of Type of Orthography Read on Brain Activity in Regular and Dyslexic Readers

**DOI:** 10.1371/journal.pone.0086016

**Published:** 2014-01-22

**Authors:** Irit Bar-Kochva, Zvia Breznitz

**Affiliations:** Edmond J. Safra Brain Research Center for the Study of Learning Disabilities, University of Haifa, Mt. Carmel, Haifa, Israel; Cuban Neuroscience Center, Cuba

## Abstract

Regular readers were found to adjust the routine of reading to the demands of processing imposed by different orthographies. Dyslexic readers may lack such adaptability in reading. This hypothesis was tested among readers of Hebrew, as Hebrew has two forms of script differing in phonological transparency. Event-related potentials were recorded from 24 regular and 24 dyslexic readers while they carried out a lexical decision task in these two forms of script. The two forms of script elicited distinct amplitudes and latencies at ∼165 ms after target onset, and these effects were larger in regular than in dyslexic readers. These early effects appeared not to be merely a result of the visual difference between the two forms of script (the presence of diacritics). The next effect of form of script was obtained on amplitudes elicited at latencies associated with orthographic-lexical processing and the categorization of stimuli, and these appeared earlier in regular readers (∼340 ms) than in dyslexic readers (∼400 ms). The behavioral measures showed inferior reading skills of dyslexic readers compared to regular readers in reading of both forms of script. Taken together, the results suggest that although dyslexic readers are not indifferent to the type of orthography read, they fail to adjust the routine of reading to the demands of processing imposed by both a transparent and an opaque orthography.

## Introduction

According to the principle of orthographic depth [1]–[2], orthographies vary from shallow orthographies with transparent grapheme-phoneme relations (*e.g.* German, Italian), to deep orthographies, in which these relations are opaque (*e.g.* English). A considerable number of behavioral studies have associated phonological decoding of small orthographic units with reading of shallow orthographies more than with reading of deep ones, and some have even associated phonological effects with reading of shallow orthographies alone. The reliance on larger orthographic units has, however, been more associated with the reading of deep orthographies [1]–[5]. These differences were suggested to reflect the distinct demands of processing imposed by shallow and deep orthographies: grapheme-phoneme conversion should be efficient in reading when grapheme-phoneme relations are transparent; however, the same procedure may be insufficient when these relations are opaque.

Only few brain imaging studies compared brain activity in reading of different orthographies; nevertheless, these corroborate the findings from the behavioral studies. In a PET study by Paulesu and his colleagues [6] localization of brain activity was compared between readers of the deep English orthography and readers of the shallower Italian orthography. English readers exhibited stronger activations than Italian readers in areas associated with irregular word reading and whole word retrieval - the left posterior inferior temporal gyrus and the anterior inferior frontal gyrus. Italians showed stronger activation in left superior temporal regions, areas associated with phoneme processing. In another study, Simon and his colleagues [7] compared event-related-potentials (ERPs) of French monolinguals and French and Arabic bilinguals when performing a lexical decision task in the two languages. When Arabic is presented without diacritics, as was done in this study, it is a deeper orthography than the French one. Only the reading of French elicited the N320 component (and in both groups), suggested to reflect spelling-to-sound conversion [8]–[9].

In a previous study we examined brain activity of regular readers of Hebrew using ERPs [10]. Speakers of Hebrew were examined, as they are skilled in reading two forms of script which transcribe the same oral language, but vary in orthographic depth. This allowed the application of a within-subject-and-language study design, which has the advantage of eliminating the possible influence of linguistic and cultural heterogeneity involved in between-language and between-subject study designs. One form of Hebrew orthography is the shallow pointed script (with diacritics), and the other is the deep unpointed script (without diacritics). The presentation of pointed and unpointed words in a lexical decision task elicited distinct brain activity at ∼165 ms and ∼340 ms after target onset. The results of the early latency, previously associated with perceptual visual-orthographic processing [8], [11]–[14], suggested that the differences may only partially be related to the different visual appearance of the two forms of script (the presence of diacritics). The latency of the next effect of form of script has been associated with orthographic-lexical processing and with the categorization of stimuli [8], [15]–[20]. These results may, therefore, reflect distinct course of visual word processing in a shallow and a deep orthography.

Notably, it was suggested that if readers of Hebrew apply distinct processing in reading the two forms of Hebrew script, then readers of other shallow and deep orthographies would, all the more so, apply different routines in reading [21]. Namely, although readers of Hebrew are skilled in reading both forms of Hebrew script, from the 5^th^ grade on they are usually more exposed to the unpointed script. Previous studies of adult skilled readers of Hebrew suggested that they applied phonological decoding of small orthographic units in reading the pointed script more than in reading the unpointed script, while addressing larger orthographic representations in reading the unpointed script [21]–[28]. These differences appeared even when unambiguous frequent words were introduced. Such words could have been easily recognized by adult skilled readers without the decoding of the diacritics. As previously suggested, the readers appeared to have changed their everyday reading routine in reading the script they were less exposed to, despite the fact that it was unnecessary [21].

The reviewed studies converge to indicate that regular readers adjust processing to the type of orthography read. The question arises whether dyslexic readers possess the same ability. Dyslexia is manifested in difficulties in accurate and/or fluent word recognition, suggested to have a neurobiological basis [29]–[30]. Behavioral data indicate that dyslexic readers stick to immature and non-optimal strategies of reading even after many years of print exposure [31]–[32]. If dyslexic readers fail to apply efficient routines in reading, even as experienced readers, their processing of print may be rigid. If so, they are expected to process orthographies of varying depth in a similar way.

Two brain imaging studies support this assumption. In another PET study by Paulesu and his colleagues [33] no differences in regions of brain activity were obtained between dyslexic readers of French, Italian and English during explicit and implicit reading. Similar brain activation was also found in an MRI study by Hu and his colleagues [34] in dyslexic readers of an alphabetic (English) and a non-alphabetic orthography (Chinese), in contrast to findings on regular readers of these orthographies.

PET and MRI brain imaging techniques have good spatial resolution but low temporal resolution. The processing of print is automatic, occurring in the range of tens to only a few hundreds of ms. Nevertheless, it is highly complex, involving a variety of sub-processes, from visual perception to higher order functions of information processing [31]. Recordings of event-related-potentials (ERPs) offer good time resolution by providing on-line information while cognitive processes are taking place. As effects of form of script read on brain activity may be revealed at different stages of processing associated with distinct aspects of processing, *e.g* visual-perceptual or linguistic stages of processing [8], the purpose of this study was to provide complementary temporal information on the relations between reading proficiency, brain activity and the reading of orthographies of varying depth. To this end, ERPs of the sample of our previous work on regular readers of Hebrew [10] was compared to a sample of dyslexic readers of Hebrew, while both groups carried out a lexical decision task in the two forms of Hebrew script.

Three amplitudes, suggesting differences in the processing of the two forms of Hebrew script at least in one group (regular/dyslexic readers) were analyzed. The first two amplitudes were the same amplitudes analyzed in our previous work on regular readers [10], *i.e.* the N170 component [8], [11]–[14] and a late central-parietal activity occurring around 340 ms after target onset [8], [15]–[20]. As dyslexic readers were expected to have slower processing of print compared to regular readers [31] the analysis was extended to a positive central-parietal amplitude occurring at ∼400 ms after target onset.

Studies have shown that dyslexic readers elicited smaller N170 amplitudes compared to regular readers when presented with orthographic stimuli, suggesting that dyslexic readers have reduced early neural specialization for orthographic stimuli [35]–[36]. Consequently, dyslexic readers may not respond differently to the presentation of the two forms of script from such an early stage. Therefore, they were expected to show lack of, or reduced effect of form of script on the N170 compared to regular readers. Later differences in brain activity elicited by the two forms of script in dyslexic readers would suggest that their neural system is responsive to the type of script presented, but their response is delayed compared to the same response in regular readers. If, however, dyslexic readers hang to a certain routine in reading, as previously suggested [32]–[34], no differences would be expected in their brain activity when processing the two forms of script either at the early or at the late amplitudes analyzed.

It should be noted that the two forms of Hebrew script differ not only in orthographic depth but also in visual load (resulting from the addition of the diacritics to the pointed script only). The N170 component was found to have distinct characteristics in response to the presentation of stimuli of different lengths, whether these were orthographic or non-orthographic [37]. Therefore, and as described in our previous work on regular readers [10], a non-orthographic decision task was administered, in addition to the lexical decision task, in an attempt to disentangle the possible effects of the visual and the orthographic differences between the two forms of Hebrew script. Non-orthographic stimuli were presented with or without invented diacritics (Table S1), and participants had to decide whether the stimuli were tilted or not. If differences in the N170 amplitude elicited by the two forms of script are of a visual source, then similar differences would be expected in the N170 component elicited by non-orthographic stimuli with and without diacritics. However, if the two forms of script elicit distinct brain activity reflecting distinct orthographic processing, then a different pattern of results should be expected when orthographic and non-orthographic stimuli with and without diacritics are compared.

## Results

### Electrophysiological Measures

**Figure 1:**
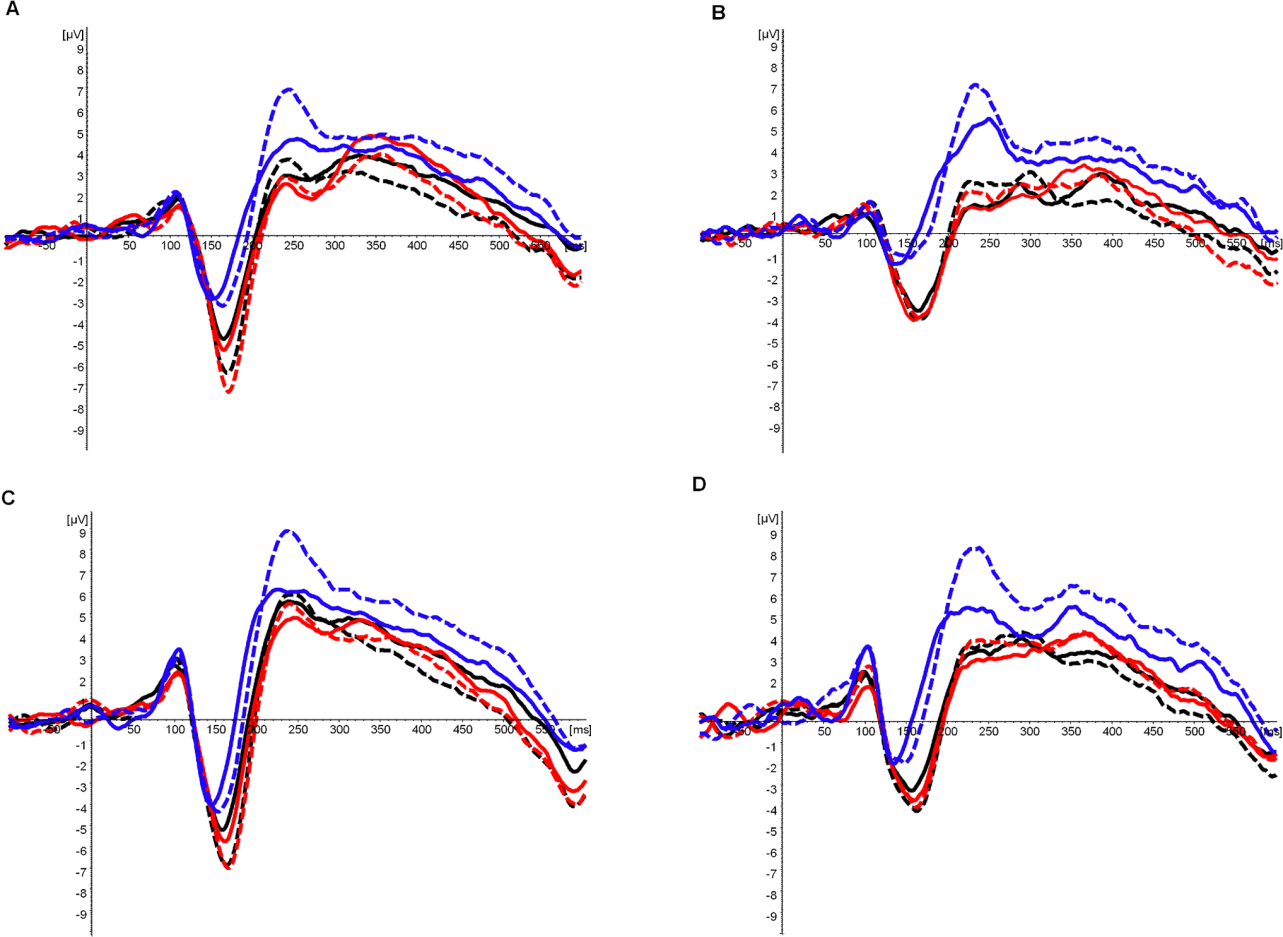
The N170 Component Elicited by the Different Stimuli. A. N170 amplitude at a left occipital-temporal electrode (PO7) in regular readers. B. N170 amplitude at a left occipital-temporal electrode (PO7) in dyslexic readers. C. N170 amplitude at a right occipital-temporal electrode (PO8) in regular readers. D. N170 amplitude at a right occipital-temporal electrode (PO8) in dyslexic readers. Continuous lines represent stimuli without diacritics and dashed lines represent stimuli with diacritics. Words are colored black, pseudowords red, and sequences of squares are in blue.

**Figure 2:**
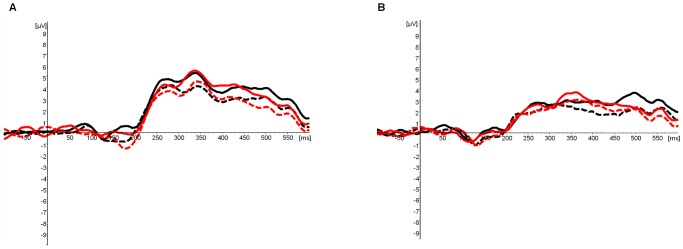
Amplitudes Elicited by the Different Stimuli around 340(P3) and 400 ms (P4). A. P3 and P4 amplitudes at a central-parietal electrode cluster (PO3, PO4 and POZ) in regular readers. B. P3 and P4 amplitudes at a central-parietal electrode cluster (PO3, PO4 and POZ) in dyslexic readers. Continuous lines represent stimuli without diacritics and dashed lines represent stimuli with diacritics. Words are colored black, pseudowords red, and sequences of squares are in blue.

**Figure 3:**
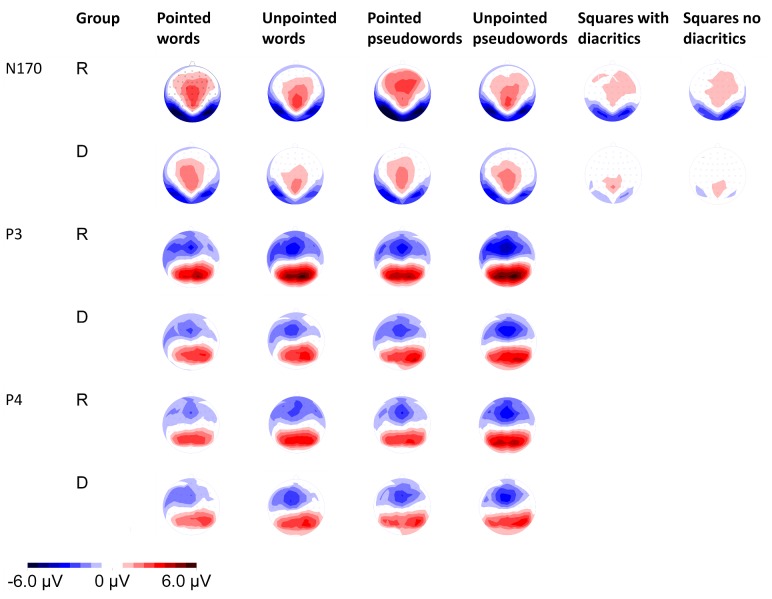
Scalp Topographies of the N170 Component and of the Amplitudes Elicited by the Different Stimuli around 340 (P3) and 400 (P4) ms. Scalp topographies in response to the presentation of words, pseudowords (N170, P3 and P4) and squares (N170) with and without diacritics. Red represents positive electrophysiological activity and blue represents negative electrophysiological activity.

The global field power based on all channels [38], the scalp distributions of each participant and the grand averages across participants were first visually inspected. The time windows analyzed were the ones suggesting differences between the two forms of script: 120–180 ms, 320–380 ms and 390–420. Within each of these time-windows, stimuli with and without diacritics showed similar topographies, while differences were observed in amplitudes and latencies at the electrodes showing maximum activity (Figures 1, 2, 3 and Tables 1–2). Presuming the reoccurring pattern of activity at these sites and latencies reflected brain activity associated with the processing of the stimuli presented, these were selected for statistical analysis [38]. In order to reduce bias associated with peak detection due to individual differences of a single point on an amplitude, the amplitudes’ strength was calculated as the mean activity recorded during 25 ms around the peaks observed.

**Table 1:** Mean Amplitudes Analyzed (in mV, Standard Deviations in Parentheses) at each Time-Window (in ms) in Regular (Reg) and Dyslexic (Dys) Readers.

			Words		Pseudowords		Squares	
Time window	Group	Electrode	Pointed	Unpointed	Pointed	Unpointed	Diacritics	No diacritics
120–180	Reg	PO7	−7.05 (5.76)	−5.17 (5.16)	−7.43 (5.52)	−5.87 (5.44)	−4.14 (4.39)	−3.72 (4.52)
	Dys		−5.31 (2.35)	−4.98 (2.57)	−5.89 (2.63)	−5.47 (2.86)	−2.27 (2.83)	−2.33 (2.99)
	Reg	PO8	−7.23 (6.04)	−5.63 (5.72)	−7.27 (5.65)	−6.09 (5.42)	−4.58 (6.44)	−3.70 (6.45)
	Dys		−5.50 (5.95)	−4.48 (5.86)	−5.08 (6.90)	−4.74 (7.24)	−2.73 (3.89)	−2.39 (3.85)
320–380	Reg	PO3, PO4,POZ	4.84 (4.69)	5.82 (4.22)	5.16 (5.07)	6.07 (4.28)	–	–
	Dys		3.67 (3.60)	3.95 (3.07)	4.04 (3.29)	4.72 (3.78)	–	–
390–420	Reg	PO3, PO4,POZ	3.22 (3.85)	3.85 (3.32)	3.34 (4.01)	4.50 (4.03)	–	–
	Dys		2.25 (3.59)	3.27 (3.48)	3.02 (3.62)	3.26 (3.46)	–	–

**Table 2:** Mean Latencies of the Amplitudes Analyzed at each Time Window (in ms, Standard Deviations in Parentheses) in Regular (Reg) and Dyslexic (Dys) Readers.

			Words		Pseudowords		Squares	
Time window	Group	Electrode	Pointed	Unpointed	Pointed	Unpointed	Diacritics	No diacritics
120–180	Reg	PO7	168.58 (11.20)	163.80 (14.34)	171.92 (10.17)	164.65 (13.27)	161.13 (14.70)	156.57 (13.86)
	Dys		164.37 (18.31)	162.23 (20.51)	165.51 (21.26)	165.36 (18.22)	155.03 (13.63)	150.22 (12.54)
	Reg	PO8	165.22 (11.29)	161.52 (12.88)	165.75 (13.34)	162.72 (10.63)	159.91 (12.13)	154.20 (12.29)
	Dys		163.98 (17.45)	161.52 (18.92)	164.45 (20.43)	163.68 (17.35)	153.74 (13.22)	149.90 (13.00)
320–380	Reg	PO3, PO4,POZ	343.87 (25.62)	338.79 (20.92)	349.93 (21.31)	341.88 (22.06)	–	–
	Dys		342.00 (22.66)	338.20 (22.47)	349.81 (21.47)	351.36 (22.63)	–	–
390–420	Reg	PO3, PO4,POZ	403.23 (12.18)	406.40 (10.79)	405.12 (11.24)	403.98 (11.39)	–	–
	Dys		405.80 (11.78)	402.27 (10.16)	404.28 (11.88)	405.94 (13.18)	–	–

#### 120–180 ms (N170)

The possible effect of the visual load imposed by the Hebrew diacritics on early brain activity was evaluated by analyzing the data from the lexical decision and the non-orthographic tasks in one Repeated Measure ANOVA analysis. Similar negative maximums were obtained at left and right occipital-temporal electrodes PO7 and PO8. Therefore, a 2×3×2×2 Repeated Measure ANOVA was carried out, with electrode (PO7/PO8), stimulus (words/pseudowords/squares) and diacritics (with/without meaningful or meaningless diacritics) as within-subject factors and group (dyslexic/regular readers) as a between-subject factor.

Amplitudes (Table 1): A main effect of type of stimulus (F_(1.16,53.25)_ = 24.83, p<.001, *η_p_^2^* = .35) was obtained. Bonferroni pair-wise comparisons, based on the estimated marginal means indicated that words (mean = −5.67, SE = .63) and pseudowords (mean = −5.98, SE = .66) evoked larger amplitudes (p<.001 in both comparisons) than squares (mean = −3.23, SE = .59). The differences between words and pseudowords were insignificant.

A main effect was also found for diacritics (F_(1,46)_ = 36.18, p<.001, *η_p_^2^* = .44), together with an interaction between diacritics and group (F_(1,46)_ = 9.71, p<.01, *η_p_^2^* = .17) and a tendency for an interaction between diacritics and stimulus (F_(2,92)_ = 2.43, p = .09, *η_p_^2^* = .05). The estimated marginal means suggested larger amplitudes evoked by stimuli with diacritics (mean = −5.37, SE = .59) than by stimuli without diacritics (mean = 4.55, SE = .57). These differences appeared to be larger in regular readers (mean with diacritics = −6.28, SE = .84, mean without diacritics = −5.03, SE = .80) than in dyslexic readers (mean with diacritics = −4.46, SE = .84, mean without diacritics = −4.07, SE = .80). The estimated marginal means also suggested that the differences between stimuli with and without diacritics tended to be larger in the case of orthographic stimuli than in the case of non-orthographic stimuli (mean pointed words = −6.27, SE = .67; mean unpointed words = −5.07, SE = .61; mean pointed pseudowords = −6.42, SE = .68; mean unpointed pseudowords = −5.54, SE = .67; mean squares with diacritics = −3.43, SE = .59; mean squares without diacritics = −3.03, SE = .60). In fact, the mean amplitudes evoked at PO7 by non-orthographic stimuli with and without diacritics in dyslexic readers were almost identical (Table 1).

Latencies (Table 2): The main effects were of electrode (F_(1,46)_ = 10.57, p<.01, *η_p_^2^* = .19), stimulus (F_ (1.19,54.88)_ = 20.89, p<.001, *η_p_^2^* = .31) and diacritics (F_(1,46)_ = 28.58, p<.001, *η_p_^2^* = .38). However, there was also an interaction between these three factors and the factor of group (F_(2,92)_ = 3.14, p<.05, *η_p_^2^* = .06). In order to further understand these data a separate analysis of the two electrodes and groups (with stimulus and diacritics as within-subject factors) was carried out. A delaying effect of stimuli with diacritics appeared to be larger in regular readers than in dyslexic readers and at both electrodes (PO7 dyslexics: F_(1,23)_ = 8.63, p<.01, *η_p_^2^* = .27; PO7 regular: F_(1,23)_ = 15.40, p≤.001, *η_p_^2^* = .40; PO8 dyslexics: F_(1,23)_ = 8.84, p<.01, *η_p_^2^* = .28; PO8 regular: F_(1,23)_ = 16.84, p≤.001, *η_p_^2^* = .42). At the same time, in dyslexic readers there was an interaction approaching significance at PO7 between diacritics and stimulus (F_(2,46)_ = 2.50, p = .09, *η_p_^2^* = .10). Planed comparisons indicated that in this group and electrode the latencies of stimuli with and without diacritics differed significantly only in the case of the non-orthographic stimuli (t_(23)_ = 3.66, p≤.001).

Notably, the effect of type of stimulus was significant in both groups and electrodes, with orthographic stimuli eliciting delayed amplitudes in comparison to non-orthographic stimuli (PO7: dyslexics F_(1.12,25.71)_ = 10.52, p<.01, *η_p_^2^* = .31; regular readers F_(1.36,31.25)_ = 13.31, p<.001, *η_p_^2^* = .37; PO8: dyslexics F_(1.14,26.26)_ = 10.61, p<.01, *η_p_^2^* = .32; regular readers F_(1.60,36.91)_ = 6.87, p<.01, *η_p_^2^* = .23). The latencies of words and pseudowords did not differ significantly in either group.

#### 320–380 ms (P3)

From this time-window on the data from the non-orthographic decision task were excluded from the analysis of the lexical decision task, as beyond the early stages of visual perception the two tasks may impose essentially different demands of processing. Maximum positive activity was observed at three adjacent parietal-occipital electrodes: PO3, PO4 and POZ. A 2×2×2 Repeated Measure ANOVA with stimulus (words/pseudowords) and form of script (pointed/unpointed) as within-subject factors and group (dyslexic/regular readers) as a between-subject factor was conducted on the mean amplitudes and latencies of this cluster of electrodes.

Amplitudes: Main effects of stimulus (F_(1,46)_ = 4.34, p<.05, *η_p_^2^* = .08) and form of script were obtained (F_(1,46)_ = 12.14, p≤.001, *η_p_^2^* = .21). The estimated marginal mean indicated larger amplitudes elicited by pseudowords (mean = 5.00, SE = .58) than by words (mean = 4.57, SE = .55), and larger amplitudes elicited by unpointed stimuli (mean = 5.14, SE = .54) than by pointed stimuli (mean = 4.43, SE = .59). At the same time, the means (Table 1) suggested similar amplitudes elicited by pointed and unpointed words in dyslexic readers. In order to test this observation, planned comparisons were carried out. These confirmed that in dyslexic readers not only was the difference between pointed and unpointed words insignificant, but so was the difference between pointed and unpointed pseudowords. In regular readers, however, differences were obtained between both pointed and unpointed words (t_(23)_ = −2.88, p<.01) and between pointed and unpointed pseudowords (t_(23)_ = −2.33, p<.05).

Latencies: A main effect of type of stimulus (F_(1,46)_ = 9.68, p<.01, *η_p_^2^* = .17) was obtained. The estimated marginal means indicated that pseudowords (mean = 348.25, SE = 2.88) elicited delayed amplitudes in comparison to words (mean = 340.72, SE = 3.13). A main effect of form of script was marginally significant (F_(1,46)_ = 4.02, p≤.05, *η_p_^2^* = .08), and the estimated marginal means suggested that the pointed script (mean = 346.40, SE = 2.95) elicited delayed amplitudes in comparison to the unpointed script (mean = 342.56, SE = 2.88). However, once again, the differences between the latencies of the amplitudes elicited by pointed and unpointed stimuli in dyslexic readers were rather small. In order to test their significance planned comparisons were carried out. The only significant difference obtained was between pointed and unpointed pseudowords, and in regular readers alone (t_(23)_ = 2.05, p≤.05).

#### 380–420 ms (P4)

A 2×2×2 Repeated Measure ANOVA with type of stimulus (words/pseudowords) and form of script (pointed/unpointed) as within-subject factors and group (regular/dyslexic readers) as a between-subject factor was conducted on the mean amplitudes of the same cluster of electrodes analyzed at the previous time-window. Notably, no clear peak was observed at this latency across participants. Nevertheless, the reason for its analysis was that the earlier time-window analyzed around 340 ms suggested more similar amplitudes elicited by pointed and unpointed stimuli in dyslexic readers than in regular readers. In order to test a possible delay in the appearance of differences in processing these stimuli in dyslexic readers at the late stages of word recognition, the earliest time-window following ∼340 ms suggesting a divergence of the amplitudes of pointed and unpointed words was examined.

Amplitudes: A main effect was obtained for form of script (F_(1,46)_ = 15.37, p<.001, *η_p_^2^* = .25), with unpointed stimuli (mean = 3.72, SE = .49) eliciting larger amplitudes than pointed stimuli (mean = 2.96, SE = .52). In order to confirm the significance of the differences within the two groups, planned comparisons were carried out. In contrast to the previous time-window analyzed, the difference between pointed and unpointed words was significant in dyslexic readers (t_(23)_ = −2.17, p<.05). In regular readers the difference between pointed and unpointed pseudowords was significant (t_(23)_ = −2.85, p<.01).

Latencies: The interaction between group, stimulus and form of script was marginally significant (F_(1,46)_ = 4.02, p≤.05, *η_p_^2^* = .08). In order to understand the direction of this interaction a separate analysis of the two groups was carried out, which indicated only a tendency of interaction between stimulus and form of script in dyslexic readers (F_(1,23)_ = 3.32, p = .08, *η_p_^2^* = .13). The means suggested delayed latencies for pointed stimuli in comparison to unpointed stimuli only in the case of words (however, these only approached significance in a planned comparison, t_(23)_ = 1.77, p = .09).

### Experimental Behavioral Measures

A 2×2×2 Repeated Measure ANOVA with stimulus (words/pseudowords) and form of script (pointed/unpointed) as within-subject factors and group (regular/dyslexic readers) as a between-subject factor was conducted on the measures of accuracy and reaction times (RTs) of the lexical decision task (Table 3).

**Table 3:** Mean Accuracy (in Percentages) and Reaction Times (RTs, in ms) of Regular and Dyslexic Readers in the Lexical Decision and the Visual Non-Orthographic Orientation Decision Tasks (Standard Deviations in Parentheses).

		Lexical decision			
		Pointed		Unpointed	
Group		Words	Pseudowords	Words	Pseudowords
Regular Readers	RT	654.76 (92.90)	687.03 (100.45)	621.29 (75.64)	676.09 (90.63)
	Accuracy	95.91 (4.53)	96.59 (5.93)	96.67 (3.96)	96.44 (6.22)
Dyslexic Readers	RT	750.02 (158.15)	836.37 (196.40)	692.82 (118.10)	804.56 (199.10)
	Accuracy	91.59 (5.49)	89.32 (10.89)	91.06 (7.17)	89.02 (11.99)
		**Non-orthographic orientation decision (squares)**			
		**With diacritics**		**Without diacritics**	
		**Straight**	**Tilted**	**Straight**	**Tilted**
Regular Readers	RT	508.78 (70.47)	–	499.25 (62.92)	–
	Accuracy	97.99 (1.85)	–	98.46 (1.80)	–
Dyslexic Readers	RT	529.69 (67.22)	–	503.42 (48.23)	–
	Accuracy	95.73 (4.16)	–	96.09 (8.05)	–

Accuracy: A main effect of group (*F*_(1,46)_ = 13.86, *p*≤.001, *η_p_^2^* = .23) was obtained, with higher accuracy rates of regular readers (mean = 96.40,*SE* = 1.17) than of dyslexic readers (mean = 90.25, *SE* = 1.17).

Response times: Main effects were found for stimulus (*F*_(1,46)_ = 73.41, *p*<.001, *η_p_^2^* = .61), form of script (*F*_(1,46)_ = 15.26, *p*<.001, *η_p_^2^* = .25) and an interaction between these factors (*F*_(1,46)_ = 7.96, *p*<.01, *η_p_^2^* = .15). The means indicated that words (mean = 679.72, *SE* = 15.99) were recognized faster than pseudowords (mean = 751.02, *SE* = 21.89), and unpointed stimuli (mean = 698.69, *SE* = 17.92) were recognized faster than pointed stimuli (mean = 732.05, *SE* = 20.39). The interaction suggested that the difference between pointed words (mean = 702.39, *SE* = 18.72) and unpointed words (mean = 657.05, *SE* = 14.31) was larger than between pointed pseudowords (mean = 761.70, *SE* = 22.51) and unpointed pseudowords (mean = 740.33, *SE* = 22.33).

There was also a main effect for group (*F*_(1,46)_ = 8.82, *p*<.01, *η_p_^2^* = .16). The means indicated that regular readers responded faster (mean = 659.79, *SE* = 26.46) than dyslexic readers (mean = 770.94, *SE* = 26.46). In addition, an interaction between stimulus and group (*F*_(1,46)_ = 11.12, *p*<.01, *η_p_^2^* = .19) suggested that the difference in RTs between words (dyslexics mean = 721.42, *SE* = 22.62; regular readers mean = 638.02, *SE* = 22.62) and pseudowords (dyslexics mean = 820.47, *SE* = 30.95; regular readers mean = 681.56, *SE* = 30.95) was larger in dyslexic readers than in regular readers.

Notably, the data from the non-orthographic task were separately analyzed. Ceiling accuracy was obtained in both groups (Table 3). Planned comparisons between the RTs for non-orthographic stimuli (straight squares only) with and without invented diacritics showed a significant difference in dyslexic readers alone (*t*_(23)_ = 3.36, *p*<.01).

## Discussion

The goal of the present study was to examine whether the shallow pointed and the deep unpointed forms of Hebrew script induce distinct brain activity in dyslexic readers, as they do in regular readers [10]. Both groups elicited distinct ERPs when presented with the two forms of Hebrew script at early and late stages of visual word processing. At the same time, brain activity of regular readers appeared to be more responsive to the form of script presented at an early visual-perceptual stage of orthographic processing [8], [11]–[14], and to respond earlier at a later stage associated with orthographic-lexical processing and with the categorization of the stimuli [8], [15]–[20].

At the early stage of processing, larger N170 amplitudes with delayed latencies were elicited at right and left occipital-temporal sites by orthographic stimuli than by non-orthographic stimuli in regular and in dyslexic readers. The N170, characterized by early occipital-temporal negativity and central positivity, was found to be the first to distinguish between orthographic strings and other classes of visually presented stimuli [8], [11]–[14]. Orthographic processing should have begun then no later than ∼165 ms after target onset in both groups.

The effect of diacritics on the amplitudes and latencies of the N170 component interacted with group, indicating larger differences between stimuli with and without diacritics in regular readers than in dyslexic readers. These results are in line with the previous studies indicating lower early neural specialization for orthographic stimuli in dyslexic readers compared to regular readers [35]–[36]. The results also suggested that the differences in amplitudes between stimuli with and without diacritics tended to be larger in the case of orthographic stimuli than in the case of non-orthographic stimuli. In fact, in dyslexic readers the mean amplitudes evoked by non-orthographic stimuli with and without diacritics at the left occipital-temporal site were almost identical. These results imply that the distinct N170 amplitudes obtained in response to the presentation of the two forms of script were not merely a consequence of the different visual appearances of the two forms of script. Consequently, distinct orthographic processing is considered. As described in more detail in our previous work on regular readers [10], phonological decoding of the Hebrew diacritics was found to be an automatic procedure [24]–[26], [28]. Moreover, there are indications of early phonological processing in reading of another shallow orthography (Italian) around the same early latency [39]–[40]. This suggests that the process of decoding the diacritics had begun early on in both groups, albeit to a different extent.

The next effect of form of script was obtained around 340 ms after target onset at a central-parietal cluster of electrodes. Although an interaction between form of script and group failed to reach significance, the data suggested that the mean amplitudes of pointed and unpointed words were very much alike at this latency in dyslexic readers. Further analysis confirmed that the differences between pointed and unpointed stimuli were insignificant in dyslexic readers in terms of both amplitudes and latencies. In contrast, the amplitudes of pointed and unpointed stimuli differed significantly in regular readers. The difference between the amplitudes of pointed and unpointed words became significant in dyslexic readers only around 400 ms after target onset. As these late latencies have been associated with orthographic-lexical processing and with the categorization of stimuli [8], [15]–[20], the results suggest delayed adjustment of lexical processing to the type of script presented in dyslexic readers compared to regular readers.

As far as the behavioral measures are concerned, dyslexic readers were less accurate and slower to respond to stimuli presented in both the pointed and unpointed forms of script compared to regular readers. These behavioral findings, together with the ERP data, suggest that the neural system of dyslexic readers failed to sufficiently adjust processing to the demands imposed by either form of script.

Another behavioral finding that should be noted is that both groups showed delayed response times to stimuli presented in the pointed script compared to the unpointed script. As mentioned in the introduction, previous work has converged to indicate that phonological decoding of small orthographic units is more involved in reading the pointed script than the unpointed one in regular readers of Hebrew [21]–[28]. As the phonologically mediated route to word recognition was suggested to be slower than the route addressing orthographic representations [41], the delayed reaction times for pointed words may have reflected the application of the longer phonologically mediated pathway in reading of pointed words more than in reading of unpointed words. At the same time, it should be taken into account that adult readers of Hebrew are exposed to the unpointed script more than to the pointed one. Consequently, the results may also reflect the longer experience with reading the unpointed script.

Yet a third possibility was suggested in our previous work on regular readers [10]. The diacritics may have imposed a visual load, resulting in delayed processing of pointed stimuli compared to unpointed stimuli. The present results suggest that this may have especially been the case with dyslexic readers who showed delayed behavioral responses not only when diacritics were presented with orthographic stimuli, but also when invented diacritic were presented with sequences of squares. This finding stands well in line with previous results from readers of other orthographies [42]–[44] by suggesting that dyslexic readers have difficulty in processing visually crowded stimuli, and that this difficulty is not restricted to the processing of orthographic information. Orthographies with crowded diacritics may, therefore, impose a source of difficulty for dyslexic readers, on top of their difficulty in processing the orthographic code.

In conclusion, the use of a brain imaging technique with a high temporal resolution indicated that the type of script read affects brain activity not only of regular readers, but also of dyslexic readers. At the same time, these effects were significantly larger in regular readers than in dyslexic readers at an early stage of processing. Later on, the results suggested a delayed difference in brain activity in response to pointed and unpointed words in dyslexic readers compared to regular readers. The current ERP data add to the previous studies comparing reading of different orthographies using MRI and PET [33]–[34] by indicating that the neural system of dyslexic readers may not be indifferent to the type of script presented, either at early visual-perceptual or at later orthographic-lexical stages of processing. Nevertheless, and in line with these previous studies, in the current study the brain activity of regular readers appeared to be more tuned to the type of script read than the brain activity of dyslexic readers. As reading performance was inferior in dyslexic readers compared to regular readers, regardless of the form of script presented, the results suggest that dyslexic readers apply a more rigid routine in reading compared to regular readers, and that they fail to fine-tune the routine of reading to the demands of processing imposed by a shallow and a deep orthography.

Our results may suggest that struggling readers of different orthographies should be provided with distinct reading intervention programs, which take into account the specific characteristics of their orthography (linguistic and visual). In addition, as a large part of the population of readers in the world is exposed to more than a single orthography, dyslexic readers may benefit from training the ability to change between routines of reading.

## Materials and Methods

### Ethics Statement

The participants gave their written informed consent to take part in the study, which was approved by the institutional review board number 09/090.

### Participants

Forty eight university students participated in the study, half were dyslexic readers and half were regular readers (12 men in each group, age range of dyslexics: 20–32 years, mean age = 24.60 years, *SD* = 3.00; age range of regular readers: 20–33 years, mean = 26.12 years, *SD* = 3.07). All were native speakers of Hebrew, right handed, with normal to corrected vision, reporting no history of attention disorders or any other neurological or emotional diagnosis. There were no significant differences between the groups in verbal and visual-spatial general ability measures (Table S2), as examined by the Similarities and Block Design sub-tests from the WAIS-III [45]. The participants were paid volunteers, who responded to ads appearing around the campus. The dyslexic readers were referred mainly by the Clinic for Learning Disabilities at the University of Haifa and by support centers for learning disabled students in colleagues in the north part of Israel. All reported a long history of reading difficulties and were diagnosed as dyslexic readers in childhood. These participants were also recognized by the clinic and the support centers as dyslexic readers, according to criteria of the Israeli Ministry of Education, which are consistent with the DSM-IV [46].

A series of background cognitive and language tests, shown to distinguish between regular and dyslexic readers [47]–[48], was administered in order to confirm the assignment of participants into one of the two reading groups. Regular readers outweighed dyslexic readers in all of these tests, as presented in Table S2. Dyslexic readers also exhibited inferior skills of decoding, fluency in reading and spelling. These differences between regular and dyslexic readers are consistent with recent scientific definitions of dyslexia [29]–[30].

### Background Tests

Visual-spatial general ability was tested by the Block Design test and verbal general ability was tested by the Similarities test, both from the WAIS-III [45]. Speed of processing was examined by two additional test from the WAIS-III: Symbol Search and Digit-Symbol. Verbal speed of processing was tested by a rapid naming test [47], [49]–[50], requiring participants to quickly name 50 letters arranged in a table of 5 rows and 10 columns. Phonological awareness was examined by a phonemic segmentation test in which the experimenter reads out pseudowords, and the participant is asked to pronounce the phonemes composing each pseudoword in the correct order [51]. A phonemic omission test was also administered requiring participants to omit one phoneme from the beginning or the middle of a pseudoword [51].

#### Reading and spelling

Oral reading of unpointed words [52]: A list of 168 unpointed words arranged in order of increasing length (1 to 5 syllables) and decreasing frequency was presented and the participants were asked to accurately read as many words as possible in 1 minute.

Oral deciphering of consonants and vowels [53]: Forty-two combinations of pointed consonants and vowels were presented which the participants were required to pronounce.

Oral reading of pointed pseudowords [54]: A list of 86 pointed pseudowords arranged in order of increasing length (1 to 5 syllables) was presented. The testing procedure was the same as in the word reading test.

Oral reading of unpointed text (The Center for Psychometric Tests, 1994): A text comprising 216 words was presented to the participants, who were asked to read quickly and accurately.

Spelling [55]: Participants were required to write 30 words of various frequencies dictated by the experimenter. All words included homophonic letters, and therefore could not be correctly spelled based on phonology alone.

### Experimental Tasks

The participants completed two computerized visual decision tasks presented using the E-Prime software [56]:

#### Lexical decision

Words and pseudowords were presented to the center of the screen, and the participants had to decide whether the stimuli were real words or pseudowords. Due to the sensitivity of ERP recordings, only certain categories of words were included: the words were unambiguous concrete nouns, 3–5 letters in length, with a single meaning. In addition, none of the words contained the Hebrew vowel letters (“*yud*”) and (“*vav*”), since in Hebrew pointed script, some of these letters are omitted and replaced by diacritics. The inclusion of such words would have created differences in the number of letters in a word when presented with or without diacritics.

A list of frequent words was compiled as elaborated in our previous work on regular readers [10], [57]–[58]. Pseudowords were created on the basis of the real words by changing one letter of each word while maintaining the word’s morphological pattern [59]–[60]. Each lexical decision task contained 55 words and 55 pseudowords.

#### Non-Orthographic orientation decision

Sequences of squares were presented with or without meaningless diacritics, as previously described [10]. The length of each sequence and its size were matched to the orthographic stimuli in the lexical decision task. The participants were asked to decide whether the sequences of squares were tilted or not. As this task was used only for the purpose of isolating the visual difference between the two forms of script, and in order to reduce the number of variables, only data on straight squares with and without diacritics were analyzed.

### Procedure

#### Task administration

ERPs were recorded while participants carried out the two decision tasks in a sound-attenuated room. The stimuli (font David 28) were projected at random to the center of the computer monitor for 400 ms. This duration was based on findings indicating that gaze duration on words among adult readers of Hebrew varies from 229 to 267 ms [61], and that lexical access progresses gradually, requiring around 300 ms [15]. Some leeway was added to take into account variation between participants. Another 1600 ms were given to respond.

Each task was preceded by four sample trials. The participants were asked to respond immediately after the presentation of each stimulus by pressing with their right hand one keyboard button for word (or straight squares), and another button for pseudowords (or tilted squares).

In order to avoid the adaptation of a default reading strategy that is suitable for both pointed and unpointed reading [62], pointed and unpointed words and pseudowords (as well as squares) were presented in separate blocks. The addition of diacritics and the order of the tasks were counterbalanced between participants.

#### EEG recording and offline analysis

Scalp EEG data was continuously recorded using a 64 channel BioSemi ActiveTwo system (BioSemi, Amsterdam, The Netherlands) and the ActiveView recording software. Pin-type electrodes were mounted on a customized BioSemi head-cap, arranged according to the extended 10–20 system. Two flat electrodes were placed on the sides of the eyes to monitor horizontal eye movement. A third flat electrode was placed underneath the left eye to monitor vertical eye movement and blinks. During the session, electrode offset was kept below 50 mV. The EEG signals were amplified and digitized with a 24 bit AD converter. A sampling rate of 2048 Hz (0.5 ms time resolution) was employed.

ERPs were analyzed offline using the Brain Vision Analyzer software. The EEG data were filtered (high: 25 Hz and low: 0.1 Hz), and referenced to the common average of all electrodes. Ocular artifacts were corrected as described previously [63]. Correct responses were divided into epochs of 100 ms pre-stimulus baseline and 1900 ms post-stimulus. Artifacts were rejected, the resulting data were baseline-corrected, and global field power (RMS) was calculated for each segment.

## Supporting Information

Table S1 **Examples of the stimuli presented in the lexical decision and the non-orthographic orientation decision tasks.**
(TIF)

Table S2 **Performance of regular and dyslexic readers in the background measures.**
(DOCX)
