# Supplementary figures and images for: Reading Proficiency and Adaptability in Orthographic Processing: An Examination of the Effect of Type of Orthography Read on Brain Activity in Regular and Dyslexic Readers
Source: PLoS One. 2014 Jan 22;9(1):e86016. doi: 10.1371/journal.pone.0086016 (PMC3899085; doi:10.1371/journal.pone.0086016)

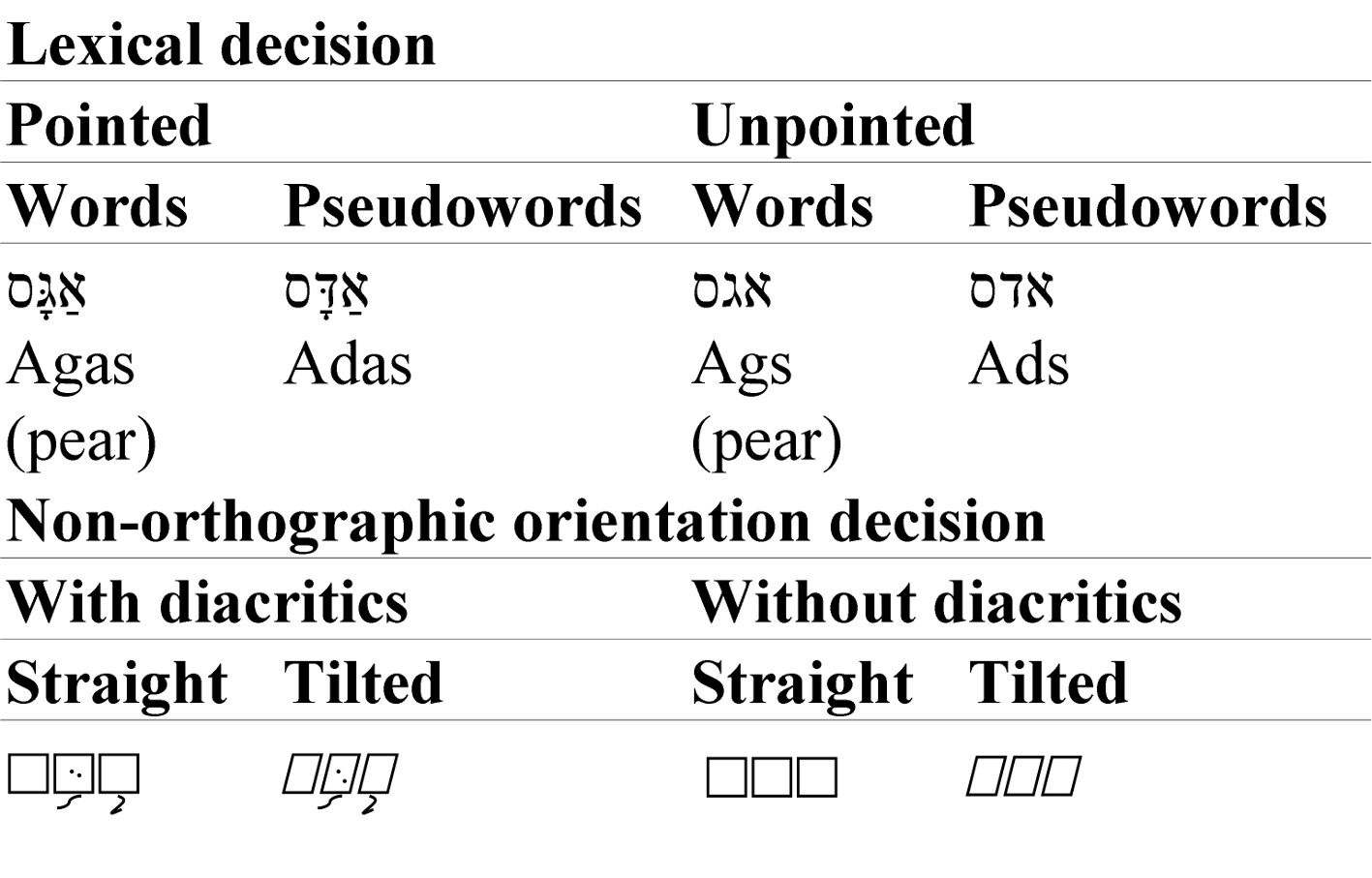

Supplement: Table S1 — Examples of the stimuli presented in the lexical decision and the non-orthographic orientation decision tasks. (TIF) [file pone.0086016.s001.tif]
