# Supplementary material for: Reading Proficiency and Adaptability in Orthographic Processing: An Examination of the Effect of Type of Orthography Read on Brain Activity in Regular and Dyslexic Readers
Source: PLoS One. 2014 Jan 22;9(1):e86016. doi: 10.1371/journal.pone.0086016 (PMC3899085; doi:10.1371/journal.pone.0086016)
Supplement: Table S2 — Performance of regular and dyslexic readers in the background measures. (DOCX) [file pone.0086016.s002.docx]

Table S2. Performance of Regular and Dyslexic Readers in the Background Measures.

|  |  | **Range** | **Mean** | **SD** | ***t*_(46)_** |
| --- | --- | --- | --- | --- | --- |
| **General Ability** |  |  |  |  |  |
| Block Design | Regular | 7-19 | 12.75 | 3.21 | ns |
|  | Dyslexics | 6-18 | 12.54 | 2.73 |  |
| Similarities | Regular | 10-18 | 12.58 | 2.53 | ns |
|  | Dyslexics | 8-17 | 11.86 | 2.31 |  |
| **Speed of Processing** |  |  |  |  |  |
| Digit Symbol | Regular | 8-15 | 11.17 | 2.24 | -2.45* |
|  | Dyslexics | 5-15 | 9.42 | 2.69 |  |
| Symbol Search | Regular | 8-18 | 11.62 | 2.58 | -3.20** |
|  | Dyslexics | 6-12 | 9.50 | 1.98 |  |
| RAN letters time (in sec) | Regular | 16-29 | 21.37 | 2.78 | *t*_(36.44)_=4.34*** |
|  | Dyslexics | 20-40 | 26.54 | 5.05 |  |
| **Phonological Awareness** |  |  |  |  |  |
| Phoneme Segmentation % acc | Regular | 43.75-100 | 93.49 | 14.45 | *t*_(37.33)_=-2.67* |
|  | Dyslexics | 6.25-100 | 77.99 | 24.44 |  |
| Phoneme Deletion % acc | Regular | 88-100 | 99.17 | 2.63 | *t*_(23.66)_=-3.29** |
|  | Dyslexics | 8-100 | 84.33 | 21.94 |  |
| **Reading and Spelling** |  |  |  |  |  |
| Words output | Regular | 95-159 | 118.58 | 14.73 | -11.96*** |
|  | Dyslexics | 39-99 | 61.83 | 17.99 |  |
| Pseudowords output | Regular | 50-84 | 68.62 | 9.62 | -13.85*** |
|  | Dyslexics | 9-48 | 26.00 | 11.62 |  |
| Consonant-Vowel output | Regular | 61.76-126 | 88.60 | 18.21 | -8.48*** |
|  | Dyslexics | 7.25-88.70 | 40.90 | 20.66 |  |
| Text output | Regular | 136.42-240 | 166.02 | 24.04 | -7.17*** |
|  | Dyslexics | 68.05-173.44 | 114.89 | 25.32 |  |
| Spelling % acc | Regular | 83.33-100 | 97.36 | 4.17 | *t*_(25.56)_=-6.38*** |
|  | Dyslexics | 30-100 | 73.75 | 17.65 |  |

Note. Acc=accuracy. Tests from the WAIS-III are presented in standard scores (Block Design, Similarities, Digit Symbol and Symbol Search). Output= correct items read within 1 minute.
